# Efficacy and safety of fluticasone furoate 100 μg and 200 μg once daily in the treatment of moderate-severe asthma in adults and adolescents: a 24-week randomised study

**DOI:** 10.1186/1471-2466-14-113

**Published:** 2014-07-09

**Authors:** Ashley Woodcock, Jan Lötvall, William W Busse, Eric D Bateman, Sally Stone, Anna Ellsworth, Loretta Jacques

**Affiliations:** 1 Institute of Inflammation and Repair, University of Manchester, University Hospital of South Manchester, Manchester M23 9LT, UK; 2 Krefting Research Centre, University of Gothenburg, Gothenburg, Sweden; 3 Department of Medicine, University of Wisconsin, Madison, WI, USA; 4 Department of Medicine, University of Cape Town, Cape Town, South Africa; 5 Respiratory Medicine Development Centre, GlaxoSmithKline, London, UK; 6 Quantitative Sciences Division, GlaxoSmithKline, RTP, Durham, NC, USA

## Abstract

**Background:**

Inhaled corticosteroids are a mainstay of therapy for persistent asthma, but suboptimal adherence with twice-daily use is widespread. Fluticasone furoate (FF) is a new inhaled corticosteroid (ICS) suitable for once-daily dosing in asthma. This study was performed to descriptively assess the efficacy and safety of two doses of FF, with no planned formal statistical hypothesis testing.

**Methods:**

This was a 24-week double-blind, multicentre, parallel-group study (NCT01431950). Patients aged ≥ 12 years with moderate-severe persistent asthma and uncontrolled on mid-high dose ICS were stratified by baseline FEV_1_ and randomised (1:1) to treatment with FF 100 μg or 200 μg once daily in the evening. The primary endpoint was change from baseline trough FEV_1_ after 24 weeks; secondary and other endpoints included peak expiratory flow (PEF) and rescue-free and symptom-free 24-hour periods over Weeks 1–24, and Asthma Control Test™ (ACT) score at Week 24. A pre-specified subgroup analysis of patients by randomisation strata was performed for the primary and selected secondary and other endpoints. Safety assessments included adverse events, laboratory and vital sign measurements, and change from baseline in 24-hour urinary cortisol at Week 24.

**Results:**

With FF 100 μg and 200 μg, least squares mean trough FEV_1_ improved from baseline by 208 mL and 284 mL, respectively, at Week 24; treatment difference: 77 mL (95% CI: –39, 192). Similar improvements from baseline in rescue- and symptom-free periods, and morning and evening PEF were observed in both groups. Patients were 42% more likely to be well-controlled (ACT score ≥ 20) with FF 200 μg than with FF 100 μg. Slightly more patients receiving FF 200 μg vs. FF 100 μg reported adverse events (63% vs. 59%) and events deemed treatment related (5% vs. <1%). Seven serious adverse events (FF 200 μg 4; FF 100 μg 3) were reported, none of which were deemed treatment related. No clinically relevant effects of either dose on 24-hour urinary cortisol were observed.

**Conclusion:**

Improvements from baseline in trough FEV_1_ were observed after 24 weeks of treatment with both doses of FF, with a numerically greater improvement in FEV_1_ observed in patients receiving FF 200 μg. Secondary endpoint findings were similar between groups. No safety concerns were identified during the study.

## Background

Asthma is a chronic inflammatory disorder that affects approximately 300 million individuals worldwide [1]. Effective maintenance therapy in asthma can improve lung function, minimise symptoms and reduce the likelihood of exacerbations that can result in hospitalisation and mortality. Anti-inflammatory medications, primarily inhaled corticosteroids (ICS) are the mainstay of maintenance treatment of all severities of asthma [2]. Despite the well-documented effectiveness of maintenance therapy in alleviating the symptoms of asthma in randomised controlled trials, non-adherence to anti-inflammatory medication is a significant problem in clinical practice, even in patients with severe asthma [3]. A number of factors have been found to contribute to non-adherence in this patient population, one of which is dosing frequency [4]. Hence, an efficacious, once-daily, inhaled ICS with a good safety profile may benefit patients and provide better outcomes in asthma management [5].

Fluticasone furoate (FF) is a new ICS in development for the treatment of asthma and–in combination with vilanterol, a new long-acting beta_2_ agonist (LABA)–for asthma and chronic obstructive pulmonary disease. FF is structurally distinct from fluticasone propionate (FP) [6] and has demonstrated a longer duration of action than FP *in vitro*[7]. In addition, an *in vitro* study of the glucocorticoid receptor binding affinity of FF compared with that of other ICS, FF displayed a markedly greater affinity and high retention in human lung tissue [8]. Once-daily dosing of FF is non-inferior to the same total dose of FF given twice daily [9], and once-daily doses of FF 100 μg and 200 μg are effective in improving lung function and asthma symptoms in 8-week studies of patients with asthma uncontrolled on low- or mid-dose ICS, with an acceptable safety profile [10,11]. Once-daily FF 200 μg has been shown to produce similar improvements in trough FEV_1_ to twice-daily FP 500 μg over 24 weeks in patients with moderate-severe asthma [12] and is considered to be high-dose ICS. Once-daily FF 100 μg has been shown to produce similar improvements in trough FEV_1_ over 24 weeks to twice-daily FP 250 μg in patients uncontrolled on low-mid dose ICS [13]. Previous data [10,14] have suggested that FF 200 μg may be more effective than FF 100 μg in improving lung function in more severely impaired patients (FEV_1_ ≤ 65% predicted). Pharmacokinetic analysis of FF has showed that the absolute bioavailability of FF is 14%, and that systemic exposure is dose proportional. In healthy subjects, there were no apparent safety issues, even at supra-therapeutic doses [15].

This was a descriptive study for which no formal hypothesis testing or statistical inference was planned. The objective of the study was to evaluate the efficacy and safety of two strengths of once-daily FF, 100 μg and 200 μg, in a moderate-severe asthma patient population (≥ 12 years of age) uncontrolled on mid-high dose ICS over a 24-week period, and to descriptively assess the possibility of a benefit of the higher strength of FF in such a patient population. No placebo group was included in this study, as it was not considered ethical to randomise patients with moderate-severe asthma to receive placebo for 24 weeks. Patients were stratified by baseline % predicted FEV_1_ at randomisation and, in a pre-specified sub-analysis, efficacy findings were compared between the randomisation strata.

## Methods

### Patients

Patients aged ≥ 12 years were eligible if they had a diagnosis of asthma as defined by NIH [16] for ≥ 12 weeks, were using ICS at any dose for ≥ 12 weeks and at a stable mid-high dose for ≥ 4 weeks prior to screening, and demonstrated best FEV_1_ of 40–90% predicted and reversibility of ≥ 12% and ≥ 200 mL with albuterol at screening. A summary of permitted and prohibited medications, from 12 weeks prior to screening and throughout the study, and study withdrawal criteria, are provided in Additional file 1.

Patients entered a 4-week run-in period, during which they used their baseline ICS at a stable dose, but stopped any non-corticosteroid controller medication. Eligible patients demonstrated evening pre-dose FEV_1_ of 40–90% predicted, and over the last 7 days of run-in had documented use of rescue medication and/or asthma symptoms, completed the daily diary measures, and complied with baseline medication on ≥ 4 days.

All patients gave written informed consent prior to the performance of any study-specific procedures. The study was approved by local ethics review committees (Additional file 2) and was conducted in accordance with the Declaration of Helsinki [17], Good Clinical Practice guidelines [18] and all applicable regulatory requirements.

### Study design and treatments

This Phase III, multicentre, randomised, double-blind, parallel-group study (GSK study number: FFA114496; clinicaltrials.gov registration number: NCT01431950) was conducted between 12 September 2011 and 03 October 2012. Twenty-seven centres in six countries (Argentina, United States, Chile, Russian Federation, Mexico, France) randomised patients.

During the run-in period, baseline safety evaluations and measures of asthma status were completed. Eligible patients were then stratified according to baseline FEV_1_ (≥ 40–≤ 65% or > 65%–≤ 90% predicted) and randomised (1:1) within their strata, in a double-blind manner, to receive one of two strengths of once-daily FF via the ELLIPTA® dry powder inhaler (DPI): FF 100 μg (representing an emitted dose from the DPI of 92 μg) or 200 μg (representing an emitted dose of 184 μg)^a^.

Patients were assigned to study treatment following a telephone call to the Registration and Medication Ordering System (RAMOS [GlaxoSmithKline, UK]) and randomised in accordance with a central randomisation schedule generated by the sponsor using a validated computerised system (RandAll [GlaxoSmithKline, UK]). Study medication was dispensed at each study visit following a telephone call to RAMOS to obtain the next blinded treatment pack number for each patient.

### Outcome measurements

The primary endpoint was change from baseline in evening pre-dose, pre-bronchodilator (trough) FEV_1_ measured in the clinic after 24 weeks of treatment.

Secondary efficacy endpoints were change from baseline in percentage of rescue-free and symptom-free 24-hour periods over the 24-week treatment period, and change from baseline in daily morning and evening peak expiratory flow (PEF) averaged over the 24-week treatment period. All secondary endpoints were assessed by twice-daily recordings on a hand-held electronic diary. Other endpoints included change from baseline in Asthma Control Test™ (ACT) score, change in percentage of patients whose asthma was controlled (ACT score ≥ 20) after 24 weeks, incidence of on-treatment severe asthma exacerbations, and incidence of on-treatment unscheduled asthma-related healthcare utilisation.

Safety endpoints included the incidence of adverse events (AEs) during the 24-week treatment period, 24-hour urinary cortisol (UC) excretion after 24 weeks of treatment, haematological and biochemical laboratory parameters, and routine liver function assessments. AEs were coded using the Medical Dictionary for Regulatory Activities to give a preferred term and system organ class.

### Statistical analysis

This was a descriptive study and no formal statistical inference was planned (i.e. no p-values were calculated, but it was planned *a priori* to calculate treatment differences with associated 95% confidence intervals [CIs] for all efficacy endpoints). The primary and secondary efficacy endpoints were analysed using an analysis of covariance (ANCOVA) model with effects due to baseline measurement, region, sex, age and treatment group. As this was designed to be a descriptive study, no adjustments for multiple comparisons were made. Two subgroup analyses of the primary treatment comparison were specified *a priori*: analysis by randomisation stratum and by run-in ICS use.

The safety population consisted of all patients randomised to study treatment who received at least one dose of study medication. The UC population comprised a subset of patients from the safety population who provided urine samples that did not have confounding factors that may have affected the interpretation of the UC results.

Randomisation of 220 patients (110 per arm) was planned to ensure that the half-width of the 95% CI for the primary endpoint of mean treatment difference in change from baseline in trough FEV_1_ was no larger than 110.1 mL, on the basis of assumptions of 5% withdrawal within the first 2 weeks of the treatment period and a standard deviation for the primary endpoint of 405 mL (estimated from previous studies).

As a consequence of site audits conducted by the study sponsor (GlaxoSmithKline) in connection with a previous study, concerns regarding the quality of data supplied by one study centre were raised. The intent-to-treat (ITT) population therefore comprised all patients in the safety population except for those recruited by this centre; it was decided to exclude these patients prior to unblinding, and therefore this decision is not considered to have affected the integrity of the analysis. The ITT population was the primary population for all efficacy analyses. The per protocol (PP) population comprised those patients in the ITT population who did not have protocol deviations considered to impact upon the primary efficacy analysis; the decision to exclude all or part of a patient’s data from the PP population was made prior to unblinding.

## Results

### Study population

**Figure 1:**
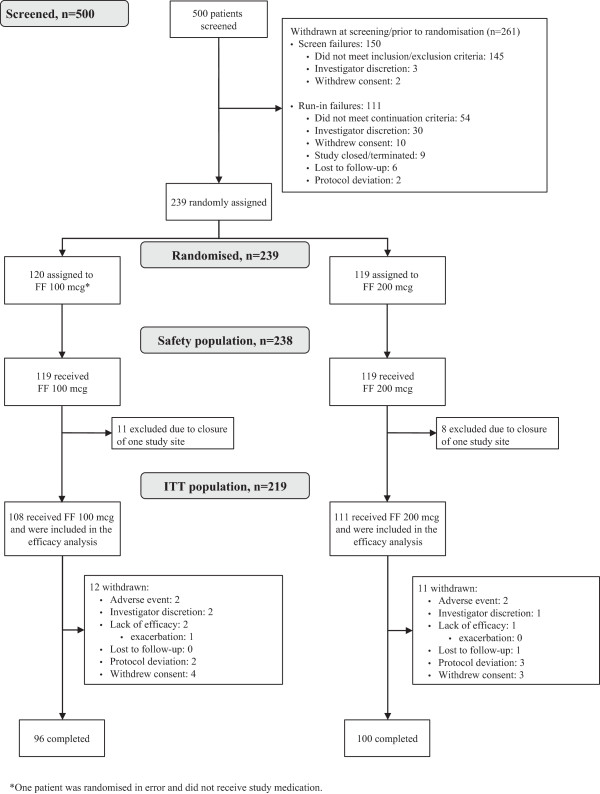
Patient disposition and reasons for withdrawal from the study.

Of 500 patients screened, 238 were randomised and received at least one dose of study medication (safety population), 219 were included in the ITT population and, of these patients, 196 (89%) completed the study (Figure 1). Adolescents (age 12–17 years) comprised 6% of the ITT population. Most (82%) patients had no history of tobacco use. Patient demographics and baseline lung function characteristics are provided in Table 1.

**Table 1:** Patient demographics and characteristics (safety population) and lung function at baseline (intent-to-treat [ITT] population)

	**FF 100 μg**	**FF 200 μg**	**Total**
**Patient demographics (safety population)**			
N	119	119	238
Age, years	46.6 (15.4)	45.1 (15.8)	45.9 (15.6)
(range)	(12–76)	(12–70)	(12–76)
Female sex, n (%)	81 (68)	79 (66)	160 (67)
White race, n (%)	101 (85)	100 (84)	201 (84)
**Patient characteristics (safety population)**			
Duration of asthma, years	20.1 (15.9)	21.5 (15.0)	20.8 (15.4)
≥ 1 exacerbation in prior 12 months, n (%)	73 (61)	67 (56)	140 (59)
**Lung function parameters (ITT population)**			
N	108*	111	219*
Percent reversibility FEV_1_ at screening,%	30.6 (16.1)	33.9 (20.6)	32.3 (18.5)
Pre-dose FEV_1_ at baseline, L	2.04 (0.67)	2.08 (0.65)	2.06 (0.66)
Percent predicted FEV_1_ at baseline,%	68.4 (14.0)	67.8 (13.3)	68.1 (13.6)

All data are mean (SD) unless otherwise stated.

*Baseline lung function data available for 107 patients in the FF 100 μg group (218 in total).

FF = fluticasone furoate; NA = not applicable; SD = standard deviation.

### Efficacy

#### Primary endpoint

**Figure 2:**
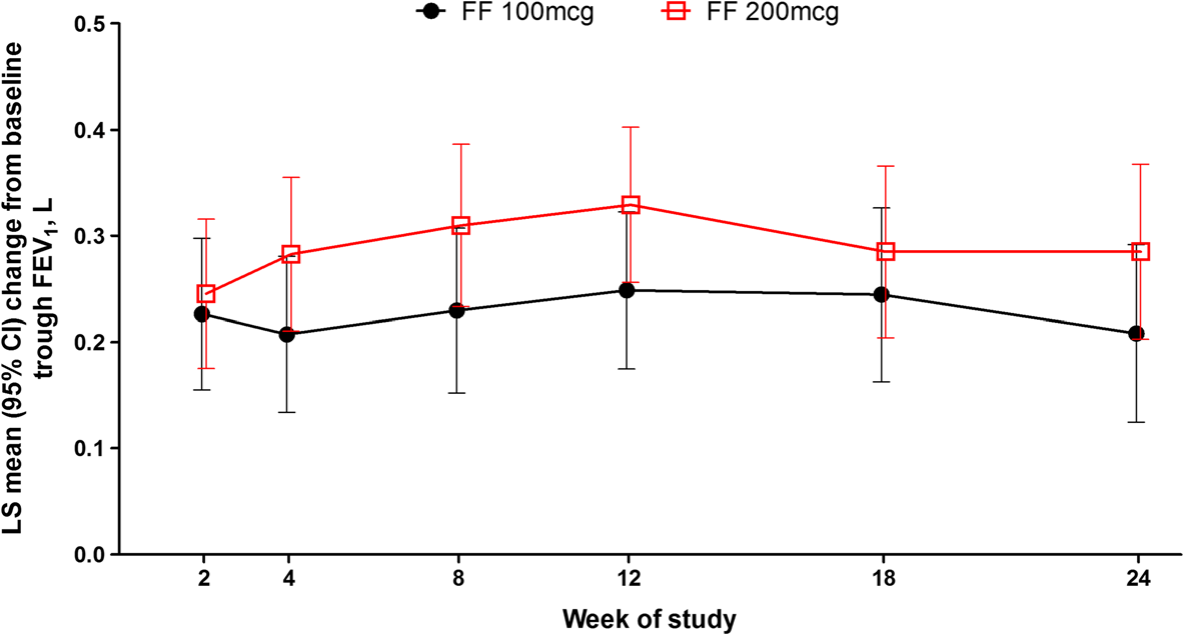
**Repeated-measures analysis of change from baseline in trough FEV**_**1 **_**(L) over 24 weeks of treatment (intent-to-treat population).** Note: repeated measures analysis adjusted for baseline, region, sex, age, treatment, visit, visit by baseline interaction, visit by treatment interaction. CI = confidence interval; FF = fluticasone furoate; LS = least squares.

Both strengths of FF were associated with improvements in trough FEV_1_ of > 200 mL from baseline at Week 24 (Table 2). A numerically greater increase was observed with FF 200 μg than with FF 100 μg (77 mL [95% CI: -39, 192]). Repeated-measures analysis of change from baseline in trough FEV_1_ over 24 weeks of treatment showed that improvement in trough FEV_1_ was apparent within 2 weeks of randomisation and was maintained throughout the treatment period (Figure 2).

**Table 2:** **Change from baseline in trough FEV**_**1 **_**(L) at Week 24 (intent-to-treat population)**

**Trough FEV**_**1 **_**(Week 24)**	**FF 100 μg**	**FF 200 μg**
	**(N = 108)**	**(N = 111)**
n	106	109
LS mean	2.271	2.347
LS mean change from baseline (SE)	0.208 (0.042)	0.284 (0.041)
Treatment difference vs.	NA	0.077
FF 100 μg (95% CI)		(-0.039, 0.192)

Note: analysis performed using ANCOVA with covariates of baseline, region, sex, age and treatment. The last observation carried forward method was used to impute missing data.

CI = confidence interval; FF = fluticasone furoate; LS = least squares; NA = not applicable; SE = standard error.

In a pre-specified subgroup analysis of the patients in each randomisation stratum (Table 3), a numerically greater increase from baseline trough FEV_1_ was observed with both FF 200 μg and FF 100 μg in the patients with FEV_1_ ≤ 65% predicted compared with those with > 65% predicted FEV_1_ at baseline. In the ≤ 65% predicted stratum, baseline FEV_1_ differed between the treatment groups (FF 200 μg: 1.699 L, FF 100 μg: 1.561 L), potentially confounding the results. A greater numerical increase at Week 24 was seen with FF 200 μg (+359 mL) compared with FF 100 μg (+305 mL) in this stratum. In the > 65% predicted stratum, in which there was no such imbalance in lung function at baseline, a greater magnitude of effect was observed with FF 200 μg (+227 mL) compared with FF 100 μg (+134 mL).

**Table 3:** **Statistical analysis of change from baseline in trough FEV**_**1 **_**(L) at Week 24 by randomisation strata and ICS dose during run-in (intent-to-treat population)**

**Trough FEV**_**1 **_**(Week 24)**	**FF 100 μg**	**FF 200 μg**
	**(N = 108)**	**(N = 111)**
**Predicted FEV**_**1 **_**at baseline**		
**≥ 40% to ≤ 65%**		
n	45	48
LS mean at baseline	1.561	1.699
LS mean at Week 24	2.368	2.422
LS mean change from baseline (SE)	0.305 (0.074)	0.359 (0.073)
Treatment difference vs.	NA	0.055
FF 100 μg (95% CI)		(-0.122, 0.232)
**> 65% to ≤ 90%**		
n	61	61
LS mean at baseline	2.387	2.372
LS mean at Week 24	2.197	2.290
LS mean change from baseline (SE)	0.134 (0.062)	0.227 (0.062)
Treatment difference vs.	NA	0.093
FF 100 μg (95% CI)		(-0.061, 0.247)
**Run-in ICS use**		
**Mid-dose**		
n	79	89
LS mean at baseline	2.039	2.085
LS mean at Week 24	2.278	2.340
LS mean change from baseline (SE)	0.222 (0.051)	0.284 (0.047)
Treatment difference vs.	NA	0.063
FF 100 μg (95% CI)		(-0.070, 0.195)
**High-dose**		
n	27	19
LS mean at baseline	2.041	1.987
LS mean at Week 24	2.228	2.360
LS mean change from baseline (SE)	0.172 (0.092)	0.304 (0.110)
Treatment difference vs.	NA	0.132
FF 100 μg (95% CI)		(-0.124, 0.388)

Note: Missing data were imputed using the last observation carried forward method.

CI = confidence interval; FF = fluticasone furoate; ICS = inhaled corticosteroid; LS = least squares; SE = standard error.

In a pre-specified subgroup analysis of patients grouped according to their run-in ICS use (mid-dose; high-dose [Additional file 1]), a greater effect on trough FEV_1_ of FF 200 μg compared with FF 100 μg was observed in those patients using high-dose ICS during run-in (Table 3). However, the size of the subgroup was small, and the 95% CI for the treatment difference was very wide and included zero.

#### Secondary and other efficacy endpoints

**Figure 3:**
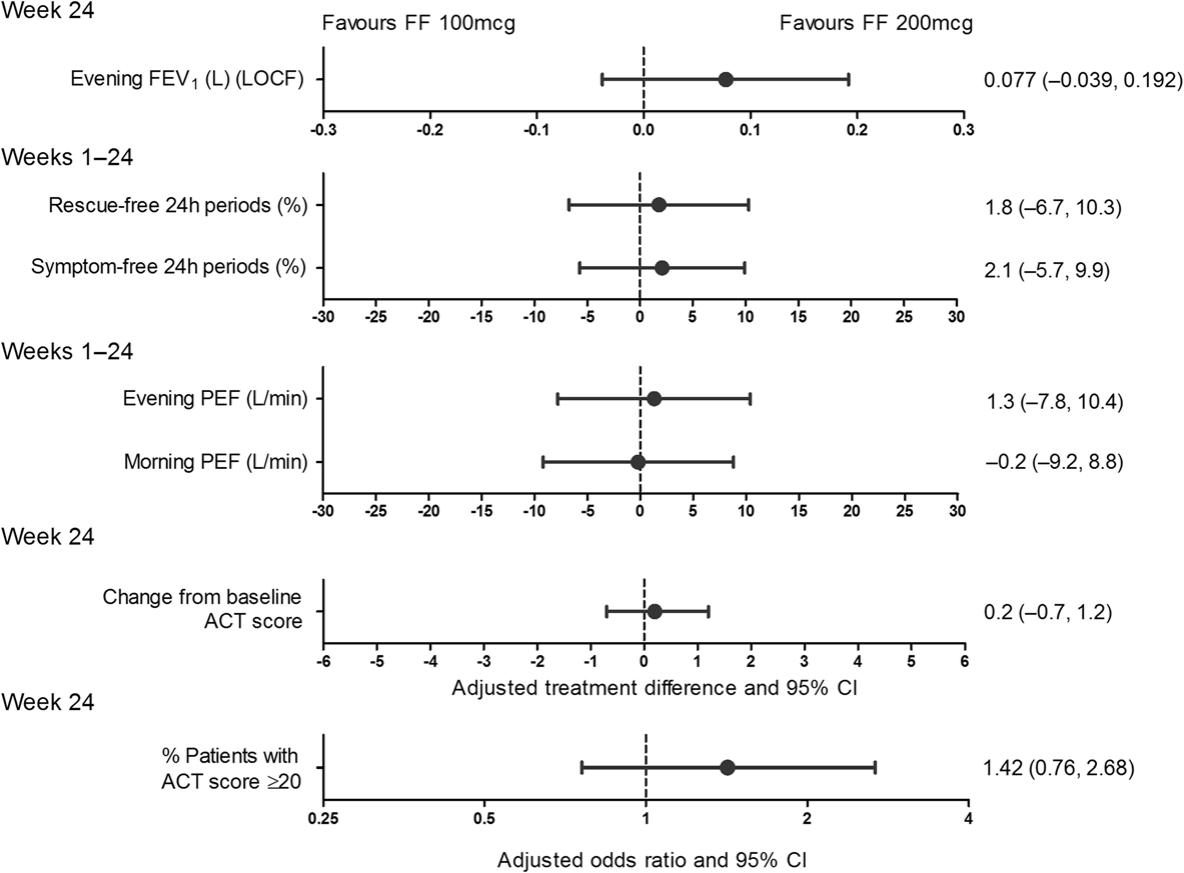
**Summary of adjusted treatment comparisons for primary, secondary and other efficacy endpoints.** ACT = asthma control test; CI = confidence interval; FF = fluticasone furoate; ITT = intent-to-treat; LOCF = last observation carried forward; OD = once daily; PEF = peak expiratory flow; PM = evening.

Observations from analysis of secondary and other endpoints supported the primary endpoint for both doses of FF (Figure 3; Table 4). Improvements over 24 weeks in percentage of rescue-free and symptom-free 24-hour periods and PEF, as well as in ACT score at Week 24, were observed in both treatment groups.

**Table 4:** Changes from baseline in secondary efficacy endpoints over Weeks 1–24 (intent-to-treat population)

	**FF 100 μg**	**FF 200 μg**
	**(N = 108)**	**(N = 111)**
**Percentage of rescue-free 24-hour periods (Weeks 1–24)**		
n	108	109
Baseline	14.3	11.5
LS mean change from baseline (SE)	21.3 (3.05)	23.1 (3.03)
Treatment difference vs. FF 100 μg (95% CI)	NA	1.8
		(-6.7, 10.3)
**Percentage of symptom-free 24**-**hour periods (Weeks 1–24)**		
n	108	109
Baseline	6.1	4.9
LS mean change from baseline (SE)	17.5 (2.80)	19.6 (2.79)
Treatment difference vs. FF 100 μg (95% CI)	NA	2.1
		(-5.7, 9.9)
**PM peak expiratory flow (L/min) (Weeks 1–24)**		
n	108	109
LS mean	342.7	344.0
LS mean change from baseline (SE)	5.9 (3.26)	7.2 (3.25)
Treatment difference vs. FF 100 μg (95% CI)	NA	1.3
		(-7.8, 10.4)
**AM peak expiratory flow (L/min) (Weeks 1–24)**		
n	108	109
LS mean	340.8	340.6
LS mean change from baseline (SE)	13.4 (3.22)	13.2 (3.20)
Treatment difference vs. FF 100 μg (95% CI)	NA	-0.2
		(-9.2, 8.8)

Note: analysis performed using ANCOVA with covariates of baseline, region, sex, age and treatment.

CI = confidence interval; FF = fluticasone furoate; LS = least squares; NA = not applicable; SE = standard error.

**Figure 4:**
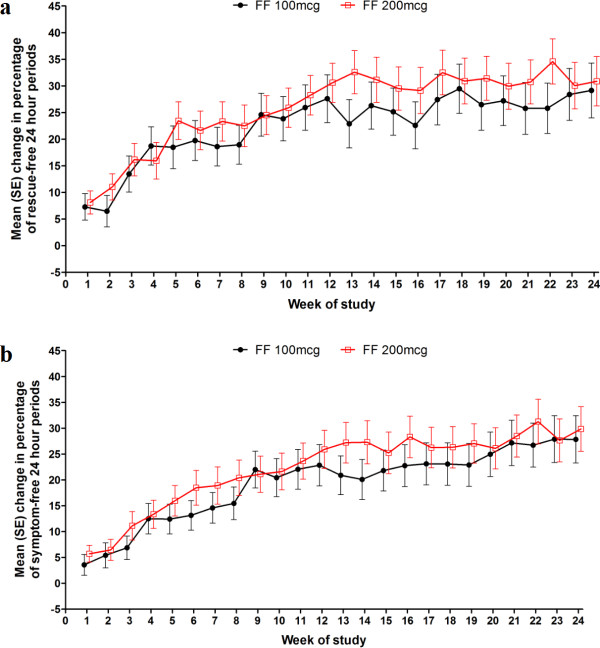
**Change from baseline in percentage of a) rescue-free 24-hour periods and b) symptom-free 24-hour periods over Weeks 0–24 (intent-to-treat population).** Note: analysis performed using ANCOVA with covariates of baseline, region, sex, age and treatment. FF = fluticasone furoate; OD = once daily; SE = standard error.

The percentage of rescue-free 24-hour periods increased over 24 weeks by > 20% in both treatment groups. Increases from baseline in rescue-free and symptom-free 24-hour periods observed in the FF 200 μg and FF 100 μg groups were, respectively, equivalent to 1.6 and 1.5 additional rescue-free days and 1.4 and 1.2 symptom-free days per week (Table 4). Change from baseline over time for these data is shown in Figure 4. Similar improvements from baseline were observed with FF 200 μg and 100 μg, respectively, for PEF (L/min) measured in the evening (7.2, 5.9) and in the morning (13.2, 13.4) (Table 4).

In both treatment groups, clinically meaningful improvement from baseline in ACT score (+ > 3.0) [19] was observed. More than half of all patients reported well-controlled asthma (ACT score ≥ 20) after 24 weeks of treatment with FF 200 μg (59%) and FF 100 μg (53%) (Odds Ratio: 1.42 [95% CI: 0.76, 2.68]), increasing from 14% in both groups at baseline.

No treatment differences were observed in incidence of severe asthma exacerbations or healthcare resource utilisation. Severe exacerbations (ATS/ERS taskforce definition [20]) were reported by 13 (12%) patients in the FF 200 μg group and 14 (13%) in the FF 100 μg group. One patient in the FF 200 μg group and two patients in the FF 100 μg group visited an emergency room because of severe asthma exacerbations. One patient in the FF 100 μg group experienced a post-treatment severe asthma exacerbation. Healthcare resource utilisation was low and similar between treatment groups: 10 patients in the FF 100 μg group and seven in the FF 200 μg group visited a physician’s office or practice. Of these, all but one patient in the FF 200 μg group reported that at least one of their physician’s office or practice visits was related to a severe asthma exacerbation. There were no asthma-related inpatient hospitalisations.

### Safety assessments

#### Adverse events and routine safety assessments

The incidence of overall AEs and treatment-related AEs was slightly higher in the FF 200 μg group than in the FF 100 μg group (Table 5). There were reports of oral or oropharyngeal candidiasis in six patients (FF 200 μg: 5, FF 100 μg: 1) and of pneumonia in one patient (FF 200 μg) that were deemed by the investigator to be related to study treatment. One patient in the FF 100 μg group experienced two post-treatment AEs (pancytopaenia and nasopharyngitis), neither of which were deemed treatment related. Four patients withdrew from the study due to an AE (FF 200 μg: cholecystitis acute and abscess, thymoma; FF 100 μg: cataract, congestive cardiomyopathy). Serious AEs were reported by four patients in the FF 200 μg group (cholecystitis acute and abscess, chondropathy, thymoma, intervertebral disc protrusion and spondylolisthesis) and three in the FF 100 μg group (chondromalacia, haemorrhagic cystitis, acute sinusitis); none was deemed by the investigator to be related to study treatment. No deaths occurred during the study. No clinically relevant treatment effects on laboratory safety tests or vital signs were observed.

**Table 5:** Summary of most frequent on-treatment AEs and serious AEs (safety population)

	**FF 100 μg**	**FF 200 μg**
	**(N = 119)**	**(N = 119)**
**AEs**		
On-treatment	70 (59)	75 (63)
On-treatment, treatment-related*	1 (< 1)	6 (5)
On-treatment, leading to withdrawal	2 (2)	2 (2)
**Serious AEs**		
On-treatment	3 (3)	4 (3)
**On-treatment AEs occurring in ≥ 5% patients in either treatment group**		
Nasopharyngitis	14 (12)	15 (13)
Headache	12 (10)	15 (13)
Bronchitis	14 (12)	8 (7)
Influenza	5 (4)	8 (7)
Sinusitis	8 (7)	5 (4)
Pharyngitis	7 (6)	4 (3)
Upper respiratory tract infection	2 (2)	7 (6)

All data are n (%).

*AEs deemed treatment-related by the investigator prior to unblinding.

AE = adverse event; FF = fluticasone furoate.

#### Urinary cortisol assessment

Least squares geometric mean 24-hour UC excretion was assessed in 148 patients (FF 200 μg: 80, FF 100 μg: 68; UC population). UC excretion at Week 24 increased from baseline in both groups. Least squares geometric mean ratios, baseline to Week 24, were 1.09 in the FF 200 μg group and 1.15 in the FF 100 μg group (FF 200 μg vs. 100 μg: 0.95 (95% CI: 0.78, 1.17). UC measurements were within the normal range at Week 24 in 95% of patients in the FF 200 μg group, and 90% in the FF 100 μg group. Shifts (baseline to Week 24) from normal or low to high urinary free cortisol were reported by three patients in the FF 200 μg group and seven in the FF 100 μg group. There were no shifts from normal or high to low, and no patients had low 24-hour urinary free cortisol excretion at Week 24.

## Discussion

FF 100 μg and 200 μg resulted in improvements from baseline in lung function and symptomatic endpoints in a population of moderate-severe asthma patients uncontrolled on mid-high dose ICS, and displayed an acceptable safety profile. The AEs seen in this study were consistent with those expected in this patient population [10,11]. There were no treatment-related serious AEs, and no safety signals of clinical concern were seen with either dose. As in previous studies testing clinical dosages of FF, alone [10] or with vilanterol [21,22], there was no evidence of cortisol suppression in patients receiving either dose.

A numerically greater effect of FF 200 μg was observed for FEV_1_ and for attainment of asthma control, with similar effects seen with both doses of FF for rescue-free and symptom-free 24-hour periods. A placebo control was not included in this study, as FF 100 μg has previously been shown to be superior to placebo in a number of studies [13,23,24], and it was not thought to be ethical for patients symptomatic on mid-high dose ICS to receive placebo for 24 weeks. The purpose of this study was to investigate whether incremental benefits of the higher FF dose could be seen in a population with a requirement for high-dose ICS. In this study, it was observed that patients using higher-dose ICS at baseline showed a comparatively greater lung function benefit with FF 200 μg than with FF 100 μg. Improvements from baseline were seen for all secondary endpoints with both FF 100 μg and FF 200 μg, with little difference between treatments for most secondary endpoints. Clinically meaningful improvement in ACT score was seen with both strengths of FF in both strata, and patients treated with FF 200 μg were 42% more likely to be well controlled (ACT score ≥ 20) after 24 weeks than those receiving FF 100 μg. However, it is important to note that none of the CIs for the primary and secondary treatment comparisons excluded the null value, and the study was not designed to statistically test for a differential treatment effect on any efficacy endpoint.

The findings of this study should be interpreted in light of the ongoing debate about the therapeutic options for patients whose asthma is uncontrolled on medium-dose ICS. Current guidelines recommend the addition of a LABA to ongoing ICS therapy in patients uncontrolled on medium-dose ICS [1]. While the addition of a LABA is generally recommended, in a subset of patients an increase in ICS dose may be more appropriate. For instance, patients whose asthma is characterised by elevated sputum eosinophil levels have been found to display substantially greater response to ICS than patients with non-eosinophilic asthma [25]. There is evidence to suggest that increasing ICS dose in such patients may be effective in reducing asthma exacerbations [26]. However, the investigation of such factors was outside the scope of the present study.

Strengths of this study include its 6-month duration and the recruitment of patients uncontrolled on mid-high dose ICS, who represent a patient population for whom the prescription of high-dose ICS maintenance therapy is appropriate. A potential limitation is that the study was not formally designed or powered to detect statistically significant treatment differences between the two treatment groups. As non-inferiority of FF 200 μg once daily to FP 500 μg twice daily has previously been demonstrated in patients uncontrolled on mid-high dose ICS [13], an active, positive-control arm was not included.

## Conclusions

Substantial improvements from baseline in lung function, asthma symptoms and patient-reported quality of life were observed after 24 weeks’ dosing with both doses of once-daily FF. Numerically greater benefit on baseline trough FEV_1_ was observed in patients receiving FF 200 μg compared with the lower dose, though this descriptive study did not incorporate testing for statistical significance. The overall safety profile of both doses of FF was good and consistent with that seen in previous studies; no safety signals of clinical concern were identified and there was no evidence of cortisol suppression.

### Endnote

^a^ELLIPTA^®^ is a trade mark of the GSK group of companies.

## Competing interests

A.W. has served as consultant to Almirall, Chiesi, Cytos and GlaxoSmithKline; and has received lecture fees and research grants from GlaxoSmithKline. J.L. has served as a consultant to, and received lecture fees from, AstraZeneca, GlaxoSmithKline, Merck Sharpe and Dohme Novartis and UCB Pharma; has been partly covered by some of these companies to attend previous scientific meetings including the ERS and the AAAAI; has provided expert testimony for Barr Pharmaceuticals and has participated in clinical research studies sponsored by AstraZeneca, GlaxoSmithKline, Merck Sharpe and Dohme, and Novartis. W.W.B. has served as a consultant to Amgen, Genentech, GlaxoSmithKline, MedImmune, and Novartis; served on advisory boards for Boston Scientific, ICON, GlaxoSmithKline and Merck Sharpe and Dohme; received research funding from the NIH; and receives royalties from Elsevier. E.D.B. has served as a consultant for Actelion, AlkAbello, Almirall, Cephalon, Hoffman la Roche, ICON, IMS Consulting Group and Navigant Consulting; been on advisory boards for Almirall, AstraZeneca, Boehringer Ingelheim, Elevation Pharma, Forest, GlaxoSmithKline, Merck, Napp, Novartis, Pfizer and Takeda; and received lecture fees from AlkAbello, AstraZeneca, Boehringer Ingelheim, Chiesi, GlaxoSmithKline, Merck, Novartis, Pfizer, Nycomed and Takeda; and received payment for development of teaching materials for Indegene Lifesciences Ltd.; and his institution has received remuneration for participation in clinical trials sponsored by Actelion, Aeras, Almirall, AstraZeneca, Boehringer Ingelheim, Cephalon, Chiesi, Forest, GlaxoSmithKline, Hoffman La Roche, Merck, Novartis, Nycomed, Takeda and TEVA. S.S., A.E., and L.J. are employees of, and hold stock in, GlaxoSmithKline.

## Authors’ contribution

WWB and AE contributed to the conception and design of the study and the analysis and interpretation of the data. AW, JL, and EDB contributed to the conception and design of the study and the interpretation of the data. SS and LJ contributed to the conception and design of the study, the writing of the study protocol and the interpretation of the data. The study was sponsored by GlaxoSmithKline. Employees of the sponsor were involved in the conception, design and conduct of the study, and in data collection and analysis. All authors, including authors employed by the sponsor, participated in the development of the manuscript, and had access to the data from the study. The decision to submit for publication was that of the authors alone. All authors read and approved the final manuscript.

## Pre-publication history

The pre-publication history for this paper can be accessed here:

http://www.biomedcentral.com/1471-2466/14/113/prepub

## Supplementary Material

Additional file 1 Online supplementary material. Click here for file

Additional file 2 List of investigational sites and IECs/IRBs FOR FFA114496. Click here for file
