# Supplementary material for: Efficacy and safety of fluticasone furoate 100 μg and 200 μg once daily in the treatment of moderate-severe asthma in adults and adolescents: a 24-week randomised study
Source: BMC Pulm Med. 2014 Jul 9;14:113. doi: 10.1186/1471-2466-14-113 (PMC4107726; doi:10.1186/1471-2466-14-113)
Supplement: Additional file 1 — Online supplementary material. [file 1471-2466-14-113-S1.docx]

**Additional file 1**

**Permitted Asthma Medications**

All patients were provided with SABA (albuterol/salbutamol inhalation aerosol) at Visit 1, which was used as needed throughout the run-in and treatment periods. Patients were not to use the albuterol/salbutamol inhalation aerosol within 6 hours prior to a clinic visit.

Patients were required to have been maintained on a stable, mid- to high-strength dose of the same ICS for the four weeks prior to Visit 1 and then throughout the run-in period. The definition of mid- and high-dose ICS medication, based on Global Initiative for Asthma (GINA, 2012) guidelines, is outlined in **Table S1**.

**Permitted Non-Asthma Medications**

The following non-asthma medications were permitted during the study:

• Decongestants

• Intranasal corticosteroids to control symptoms of allergic disorders

• Immunotherapy as long as it was initiated 4 weeks prior to Visit 1 and patients remained in the maintenance phase for the duration of the study

• Topical corticosteroids (≤1% hydrocortisone cream) for dermatological diseases

• Short-acting and long-acting antihistamines to control symptoms of allergic disorders

• Antihistamine eye drops.

**Prohibited Asthma Medications**

The following asthma medications were prohibited during the time period specified:

*Within 12 weeks of Visit 1 and during the study*

• Anti-IgE (e.g., Xolair)

• Immunosuppressive medications (immunotherapy for the treatment of allergies was permitted during the study provided it was initiated at least 4 weeks prior to Visit 1 and the patient remained in the maintenance phase throughout the study).

*Within 4 weeks of Visit 1 and during the study*

• Systemic (parenteral, oral or depot) corticosteroids. Prednisone/prednisolone was allowed during the study only for the treatment of severe asthma exacerbations

• Anticholinergics (e.g., ipratropium, tiotropium).

*Within one day of Visit 1 and during the study*

• Long-acting beta_2_-agonists (oral, inhaled or transdermal, e.g., salmeterol, formoterol, bambuterol).

• Combination products containing inhaled long-acting beta_2_-agonists (e.g., fluticasone propionate/salmeterol, budesonide/formoterol).

• Theophyllines (e.g., aminophylline).

• Leukotriene modifying agents (e.g., leukotriene receptor antagonists, montelukast, zafirlukast).

• Ketotifen

• Nedocromil sodium

• Sodium cromoglycate.

*Up to and including the morning of Visit 2 (randomisation)*

• Inhaled corticosteroids: Patients were to have been maintained on a stable, mid- to high-strength dose for four weeks prior to Visit 1 and throughout the run-in period. However, these medications were discontinued after the morning of Visit 2 with no doses taken for the remainder of the study (until end of treatment period).

*Within 6 hours prior to a study visit*

• Albuterol/salbutamol.

**Prohibited Non-Asthma Medications**

The following non-asthma medications were prohibited during the time periods specified:

*Within 4 weeks of Visit 1 and during the study*

• Potent cytochrome P450 3A4 (CYP3A4) inhibitors (e.g., ketoconazole, ritonavir, itraconazole, clarithromycin)

*From Visit 1 and during the study:*

• Any prescription or over-the-counter medication which would significantly affect the course of asthma or affect ICS metabolism

• Oral, systemic or transdermal beta-adrenergic blocking agents.

**Withdrawal criteria**

Premature discontinuation from the study occurred when a subject discontinued prior to the follow-up visit, either voluntarily or was withdrawn by the investigator. Once a subject was withdrawn from investigational product, they were considered withdrawn from the study.

Subject withdrawal was required if:

• Three protocol-defined severe asthma exacerbations were experienced within the 6 month treatment period

• A change occurred in asthma status which required the subject to be treated with maintenance asthma therapy other than the study medication or study-supplied albuterol/salbutamol

• The liver chemistry threshold was met

• An adverse event was reported that would, in the investigator’s judgment, make continued participation in the study an unacceptable risk

• The treatment blind was broken for the subject by site personnel

• The study was discontinued by the study sponsor

• A subject became pregnant.

**Supplementary Table**

**Table S1.** Daily inhaled corticosteroid (ICS) doses defined as medium- and high-dose ICS medication during run-in

| **ICS Medication** | **Medium Daily Dose** | **High Daily Dose** |
| --- | --- | --- |
| Fluticasone propionate | >250–500 µg | >500–1000 µg |
| Beclomethasone dipropionate | >500–1000 µg | >1000–2000 µg |
| Budesonide | >400–800 µg | >800–1600 µg |
| Flunisolide | >1000–2000 µg | >2000 µg |
| Triamcinolone acetonide MDI | >1000–2000 µg | >2000 µg |
| Mometasone furoate DPI | >400–800 µg | >800–1200 µg |
| Ciclesonide HFA MDI | >160–320 µg | >320–1280 µg |

DPI = dry powder inhaler; HFA = hydrofluoroalkane; ICS = inhaled corticosteroid; MDI = metered dose inhaler
