# Supplementary material for: Efficacy and safety of fluticasone furoate 100 μg and 200 μg once daily in the treatment of moderate-severe asthma in adults and adolescents: a 24-week randomised study
Source: BMC Pulm Med. 2014 Jul 9;14:113. doi: 10.1186/1471-2466-14-113 (PMC4107726; doi:10.1186/1471-2466-14-113)
Supplement: Additional file 2 — List of investigational sites and IECs/IRBs FOR FFA114496. [file 1471-2466-14-113-S2.pdf]

LIST OF INVESTIGATIONAL SITES AND IECs/IRBs FOR FFA114496

**Description of Research Facility, Hospital/ Institution, and Address**	**Name of IEC/INSTITUTIONAL REVIEW BOARD Committee, Address, Committee Chair**
**Argentina**	
INSARES, José Rondeau 335, Mendoza, Mendoza, M5500CCG, Argentina	Fundación CIDEA, Billinghurst 1677, CABA, C1425DTG, Argentina<br><br>Chairperson: Díaz Couselo, Fernando A.
Centro Médico Dra De Salvo, Avenida Cabildo 1548 1°A, Buenos Aires, C1426ABO, Argentina	Comité Independiente de Ética para Ensayos en Farmacología Clínica del Centro Médico Dra. De Salvo<br><br>Chairperson: Miranda, Adriana

Description of Research Facility, Hospital/ Institution, and Address	Name of IEC/INSTITUTIONAL REVIEW BOARD Committee, Address, Committee Chair
CIDEA, 3er Cuerpo - 2do Subsuelo, Paraguay 2035, Buenos Aires, C1121ABE, Argentina	CEIFyC – Comité de Ética en Investigación Clínica de La Federación de Círculos Católicos de Obreros y Fundación CIDEA<br><br>Chairperson: Díaz Couselo, Fernando A.
Investigaciones en Patologias Respiratorias, Balcarce 874, San Miguel de Tucumán, Tucumán., 4000, Argentina	CIEM NOA, 4th floor Las Piedras 496, San Miguel de Tucuman, Tucumán, Argentina<br><br>Chairperson: Zarba, Juan
Instituto InAER, Arenales 3146 - Piso 1 Depto B, Buenos Aires, C1425BEN, Argentina	Instituto InAER, Arenales 3146 - Piso 1 Depto B, Buenos Aires, C1425BEN, Argentina<br><br>Chairperson: Cerezo, Gustavo

**Description of Research Facility, Hospital/ Institution, and Address**	**Name of IEC/INSTITUTIONAL REVIEW BOARD Committee, Address, Committee Chair**
**Chile**	
Hospital Carlos Van Buren, San Ignacio 725, Valparaiso, Valparaíso, 2341131, Chile	Comité de Evaluación Ético Científico, Quinto Piso, Avenida Brasil #1435, Valparaíso, Chile<br><br>Chairperson: Weiss, Fernando
CIMER - Centro de Investigaciones Médicas Respiratorias, José Manuel Infante 620 - Providencia, Santiago, Región Metro De Santiago, 7500800, Chile	Comité de Etica Cientifico del Servicio de Salud Metropolitano Oriente, Av. Salvador 364, Providencia / Santiago, 7500922, Chile<br>Chairperson: Stuardo, Andrés
Hospital Dr Sotero del Rio, Av. Concha y Toro 3459, Puente Alto - Santiago, Región Metro De Santiago, 8207257, Chile	Comité de Evaluación Ético Científico SSMSO, Av. Concha y Toro #3459 - Puente Alto, Santiago, 8207257, Chile<br>Chairperson: Michaud, Patricio

**Description of Research Facility, Hospital/ Institution, and Address**	**Name of IEC/INSTITUTIONAL REVIEW BOARD Committee, Address, Committee Chair**
**France**	
CM51000 Body, Cabinet Médical, 10 quai Eugène Perret, Chalons en Champagne, 51000, France*	CPP Nord Ouest I - CHU de Rouen, Pavillon de l'Aubette, 1 rue de Germont, Rouen Cedex, 76031, France<br><br>Chairperson: Delangre, Thierry
CM44300 Boye, Cabinet Médical, 63, rue de la Bottière, Nantes, 44300, France*	CPP Nord Ouest I - CHU de Rouen, Pavillon de l'Aubette, 1 rue de Germont, Rouen Cedex, 76031, France<br><br>Chairperson: Delangre, Thierry

Description of Research Facility, Hospital/ Institution, and Address	Name of IEC/INSTITUTIONAL REVIEW BOARD Committee, Address, Committee Chair
CM44140 Hulot De Collart, Cabinet Médical, 6 rue de la Filée, Aigrefeuille Sur Maine, 44140, France	CPP Nord Ouest I - CHU de Rouen, Pavillon de l'Aubette, 1 rue de Germont, Rouen Cedex, 76031, France<br><br>Chairperson: Delangre, Thierry
CM02000 Klink, Cabinet Médical, 2 bis rue Gabriel Péri, Laon, 02000, France*	CPP Nord Ouest I - CHU de Rouen, Pavillon de l'Aubette, 1 rue de Germont, Rouen Cedex, 76031, France<br><br>Chairperson: Delangre, Thierry
CM35890 Lemarie, Cabinet Médical, 8, rue des Nouettes, Bourg Des Comptes, 35890, France*	CPP Nord Ouest I - CHU de Rouen, Pavillon de l'Aubette, 1 rue de Germont, Rouen Cedex, 76031, France<br>Chairperson: Delangre, Thierry

CM56000 Lemoine, Cabinet Médical,<br>15, Place de la République,<br>Vannes, 56000, France*	CPP Nord Ouest I - CHU de<br>Rouen, Pavillon de l'Aubette, 1<br>rue de Germont, Rouen Cedex,<br>76031, France<br>Chairperson: Delangre, Thierry

**Description of Research Facility, Hospital/ Institution, and Address**	**Name of IEC/INSTITUTIONAL REVIEW BOARD Committee, Address, Committee Chair**
**Mexico**	
Hospital del Niño de Alta<br>Especialidad, Centro de Asma,<br>Méndez 2832 Colonia Tamulté,<br>Villahermosa, Tabasco, 86100,<br>Mexico	Comité Bioético para la<br>Investigación Clínica S.C., Puebla<br># 422-4 Colonia Roma, Mexico<br>City, 06700, Mexico<br><br>Chairperson: Ovadia-Savariego,<br>Celia
Hospital Angeles Lindavista, Torre<br>de Consultorios, Río Bamba 639 Col.<br>Magdalena de las Salinas, Mexico<br>City, 07760, Mexico	Comite Bioetico para la<br>Investigación Clínica, Puebla #<br>422 -4, Colonia Roma, 07760,<br>Mexico<br><br>Chairperson: Ovadia-Savariego,<br>Celia

Description of Research Facility, Hospital/ Institution, and Address	Name of IEC/INSTITUTIONAL REVIEW BOARD Committee, Address, Committee Chair
**Russian Federation**	
Kazan Research Institute of Epidemiology and Microbiology MoH RF, 67 Bolshaya Krasnaya ul., Kazan, 420015, Russian Federation	Kazan Scientific Research Institution of Epidemiology and Microbiology of Rospotrebnadzor, 67, Bolshaya Krasnaya Street Kazan, 420015, Russian Federation Chairperson:Andreev, Sergey
Municipal children hospital 4, 33, Dimitrova street, Novokuznetsk, 654063, Russian Federation	Novokuznetsk Municipal Children Hospital 4, 33, Dimitrova street, 654063, Novokuznetsk, Russian Federation Chairperson: Domanskaya, Olga

**Description of Research Facility, Hospital/ Institution, and Address**	**Name of IEC/INSTITUTIONAL REVIEW BOARD Committee, Address, Committee Chair**
City Hospital #2, Pulmonolgy department, Admiralskogo street, 6, Pyatigorsk, 357538, Russian Federation	Ethics Committee of City Hospital #, 6, Admiralskogo Street, Pyatigorsk, 357538, Russian Federation<br><br>Chairperson: Temirov, Igor
Penza City Clinical Hospital #4, 1, Svetlaya street, Penza, 440067, Russian Federation	Penza Institute of Advanced Medical Studies, 8a, Stasova street, Penza, 440060, Russian Federation<br><br>Chairperson: Iskenderov, Bakhram

Description of Research Facility, Hospital/ Institution, and Address	Name of IEC/INSTITUTIONAL REVIEW BOARD Committee, Address, Committee Chair
**United States**	
Clinical Research Institute, Inc., Suite 435, 2805 Campus Drive, Plymouth, Minnesota, 55441, United States	Quorum Review Institutional Review Board, Suite 1000, 1601 Fifth Avenue, Seattle, 98101, United States<br><br>Chairperson: Simpson, Philip

Description of Research Facility, Hospital/ Institution, and Address	Name of IEC/INSTITUTIONAL REVIEW BOARD Committee, Address, Committee Chair
Georgia Pollens Clinical Research Center, Inc., 105 Spanish Ct., Albany, Georgia, 31707, United States	Quorum Review Institutional Review Board, Suite 1000, 1601 Fifth Avenue, Seattle, 98101, United States<br><br>Chairperson: Simpson, Philip
IPS Research Company, 1111 North Lee, Suite 400, Oklahoma City, Oklahoma, 73103, United States	Quorum Review Institutional Review Board, Suite 1000, 1601 Fifth Avenue, Seattle, 98101, United States<br><br>Chairperson: Simpson, Philip

Description of Research Facility, Hospital/ Institution, and Address	Name of IEC/INSTITUTIONAL REVIEW BOARD Committee, Address, Committee Chair
Clinical Research Specialist, Building 6, Suite 20, 3939 Houma Boulevard, Metairie, Louisiana, 70006, United States	Quorum Review Institutional Review Board, Suite 1000, 1601 Fifth Avenue, Seattle, 98101, United States<br><br>Chairperson: Simpson, Philip
Ocean Allergy & Respiratory Research Center, 1673 Highway 88 W, Brick, New Jersey, 08724, United States*	Quorum Review Institutional Review Board, Suite 1000, 1601 Fifth Avenue, Seattle, 98101, United States<br><br>Chairperson: Simpson, Philip
Community Research of South Florida, 7100 West 20th Avenue, Hialeah, Florida, 33016, United States*	Quorum Review Institutional Review Board, Suite 1000, 1601 Fifth Avenue, Seattle, 98101, United States<br>Chairperson: Simpson, Philip

Description of Research Facility, Hospital/ Institution, and Address	Name of IEC/INSTITUTIONAL REVIEW BOARD Committee, Address, Committee Chair
Allergy and Respiratory Center, 4048 Dressler Road NW, Canton, Ohio, 44718, United States	Quorum Review Institutional Review Board, Suite 1000, 1601 Fifth Avenue, Seattle, 98101, United States<br><br>Chairperson: Simpson, Philip
New Horizons Clinical Research, LLC, 4260 Glendale Milford Road Suite 201, Cincinnati, Ohio, 45242, United States	Quorum Review Institutional Review Board, Suite 1000, 1601 Fifth Avenue, Seattle, 98101, United States<br><br>Chairperson: Simpson, Philip

Description of Research Facility, Hospital/ Institution, and Address	Name of IEC/INSTITUTIONAL REVIEW BOARD Committee, Address, Committee Chair
St Elizabeth Childrens Health Center, 2212 Genesee Street, Utica, New York, 13502, United States*	Quorum Review Institutional Review Board, Suite 1000, 1601 Fifth Avenue, Seattle, 98101, United States<br><br>Chairperson: Simpson, Philip
Southern California Clinical Trials, Suite 111, 1501 Superior Ave, Newport Beach, California, 92663, United States	Quorum Review Institutional Review Board, Suite 1000, 1601 Fifth Avenue, Seattle, 98101, United States<br><br>Chairperson: Simpson, Philip
Corsicana Medical Research, PLLC, Suite #165, 301 Hospital Drive, Corsicana, Texas, 75110, United States*	Quorum Review Institutional Review Board, Suite 1000, 1601 Fifth Avenue, Seattle, 98101, United States<br>Chairperson: Simpson, Philip

**Description of Research Facility, Hospital/ Institution, and Address**	**Name of IEC/INSTITUTIONAL REVIEW BOARD Committee, Address, Committee Chair**
Allergy and Asthma Care Center of Southern CA, Suite 209, 3816 Woodruff Avenue, Long Beach, California, 90808, United States*	Quorum Review Institutional Review Board, Suite 1000, 1601 Fifth Avenue, Seattle, 98101, United States<br><br>Chairperson: Simpson, Philip
Clinical Research Consortium, Suite 103, 4275 South Burnham, Las Vegas, Nevada, 89119, United States	Quorum Review Institutional Review Board, Suite 1000, 1601 Fifth Avenue, Seattle, 98101, United States<br><br>Chairperson: Simpson, Philip
Asthma & Allergy Associates, PC, 2709 North Tejon Street, Colorado Springs, Colorado, 80907, United States	Quorum Review Institutional Review Board, Suite 1000, 1601 Fifth Avenue, Seattle, 98101, United States<br>Chairperson: Simpson, Philip

**Description of Research Facility, Hospital/ Institution, and Address**	**Name of IEC/INSTITUTIONAL REVIEW BOARD Committee, Address, Committee Chair**
Gordon D. Raphael, MD, Suite 202, 4915 Auburn Avenue, Bethesda, Maryland, 20814,United States	Quorum Review Institutional Review Board, Suite 1000, 1601 Fifth Avenue, Seattle, 98101, United States<br><br>Chairperson: Simpson, Philip
Toledo Institute of Clinical Research 7247 West Central Avenue, Toledo, Ohio, 43617, United States*	Quorum Review Institutional Review Board, Suite 1000, 1601 Fifth Avenue, Seattle, 98101, United States<br><br>Chairperson: Simpson, Philip

**Description of Research Facility, Hospital/ Institution, and Address**	**Name of IEC/INSTITUTIONAL REVIEW BOARD Committee, Address, Committee Chair**
Sunset Medical Research, 990 Napoleon Avenue, Sunset, Louisiana, 70584, United States*	Quorum Review Institutional Review Board, Suite 1000, 1601 Fifth Avenue, Seattle, 98101, United States<br><br>Chairperson: Simpson, Philip
Carolina Research, 2227 Saint Matthews Road, Orangeburg, South Carolina, 29118, United States*	Quorum Review Institutional Review Board, Suite 1000, 1601 Fifth Avenue, Seattle, 98101, United States<br><br>Chairperson: Simpson, Philip
Innovative Research of West Florida, Inc., 1573 South Fort Harrison Avenue, Clearwater, Florida, 33756, United States	Quorum Review Institutional Review Board, Suite 1000, 1601 Fifth Avenue, Seattle, 98101, United States<br>Chairperson: Simpson, Philip

Description of Research Facility, Hospital/ Institution, and Address	Name of IEC/INSTITUTIONAL REVIEW BOARD Committee, Address, Committee Chair
Allergy and Asthma Physicians of Central KY, Suite 150, 166 Pasadena Drive, Lexington, Kentucky, 40503, United States*	Quorum Review Institutional Review Board, Suite 1000, 1601 Fifth Avenue, Seattle, 98101, United States<br><br>Chairperson: Simpson, Philip

*No Subjects Enrolled

All centres participated in the study under the US IND.
